# Does preservation of the sub-valvular apparatus during mitral valve replacement affect long-term survival and quality of life? A Microsimulation Study

**DOI:** 10.1186/1749-8090-3-17

**Published:** 2008-04-23

**Authors:** Christopher Rao, Jonathan Hart, Andre Chow, Fotios Siannis, Polyxeni Tsalafouta, Bari Murtuza, Ara Darzi, Frank C Wells, Thanos Athanasiou

**Affiliations:** 1 Department Biosurgery and Surgical Technology, Imperial College London, London, UK; 2 Department of Cardiothoracic Surgery, Papworth Hospital, Cambridge, UK; 3 Medical Research Council Biostatistics Unit, Medical Research Council, Cambridge, UK

## Abstract

**Background:**

Techniques to preserve the sub-valvular apparatus in order to reduce morbidity and mortality following mitral valve replacement have been frequently reported. However, it is uncertain what impact sub-valvular apparatus preservation techniques have on long-term outcomes following mitral valve replacement. This study investigated the effect of sub-valvular apparatus preservation on long-term survival and quality of life following mitral valve replacement.

**Methods:**

A microsimulation model was used to compare long-term survival and quality-adjusted life years following mitral valve replacement after conventional valve replacement and sub-valvular apparatus preservation. Probabilistic sensitivity analysis and alternative analysis were performed to investigate uncertainty associated with the results.

**Results:**

Our Analysis suggests that patients survive longer if the sub-valvular apparatus are preserved (65.7% SD 1.5%, compared with 58.1% SD 1.6% at 10 years). The quality adjusted life years gained over a 10 year period where also greater after sub-valvular apparatus preservation. (6.54 QALY SD 0.07 QALY, compared with 5.61 QALY, SD 0.07 QALY). The superiority of preservation techniques was insensitive to patient age, parameter or model uncertainty.

**Conclusion:**

This study suggests that long-term outcomes may be improved when the sub-valvular apparatus are preserved. Given the lack of empirical data further research is needed to investigate health-related quality of life after mitral valve replacement, and to establish whether outcomes differ between preservation techniques.

## Background

The optimum management of mitral valve insufficiency is valve repair [1]. Often valve replacement is necessary, however, as repair is impossible because of anatomical or aetiological considerations [2,3].

The first Mitral Valve Replacement (MVR) involved implantation of a Starr-Edwards prosthetic valve following complete excision of the mitral leaflets, chordae tendinae and the heads of the papillary muscles [4]. Initial experience with MVR was complicated by an increased incidence of low cardiac output syndrome and associated morbidity and mortality. Subsequently, several strategies were implemented to improve postoperative outcomes, including Sub-valvular Apparatus Preservation (SVP) [5].

The concept of SVP is more than 40 years old [6-8]. Despite the publication of several studies since the late-1970s suggesting that left ventricular function and mortality were improved following SVP, particularly in patients with mitral regurgitation, it is sometimes not undertaken [5,9-29]. Whilst technical considerations may limit adoption of SVP [30], surgical strategies to overcome these technical pitfalls have been discussed in the literature in some depth [5]. Arguably uncertainty about the long-term impact of SVP on patient-focused outcomes such as free of event survival and quality of life may be a factor.

Given the body of evidence suggesting that MVR is more effective with SVP, and that in many cases SVP was technically feasible [5] we felt it was important to quantify;

1. What effect does SVP has on long-term survival?

2. Does SVP affect long-term Health-Related Quality of Life (HRQoL)?

In order to address these questions we use a microsimulation model to combine recently published mortality data from the systematic review by Athanasiou et al [5], Government Actuarial Department Data on baseline population mortality [31], valve related mortality from the United Kingdom Heart Valve Registry (UKHVR) [32] and estimates of HRQoL from this study.

## Methods

Markov microsimulation is a powerful tool that can be used to model morbidity and mortality following surgical interventions in the absence of empirical follow-up data [33,34]. It has been widely used in both general medical [35-38] and cardiothoracic journals [39-43] and is often a fundamental element of national technology assessment programmes [33,34,44-46]. It has previously been used to model outcomes following aortic valve replacement [40-43].

In microsimulation it is assumed that a patient's HRQoL can be described by a finite number of states, and by modelling the transition between these states at the end of discrete time periods, called cycles, long term predictions can be made about HRQoL and survival.

### Microsimulation model

**Figure 1:**
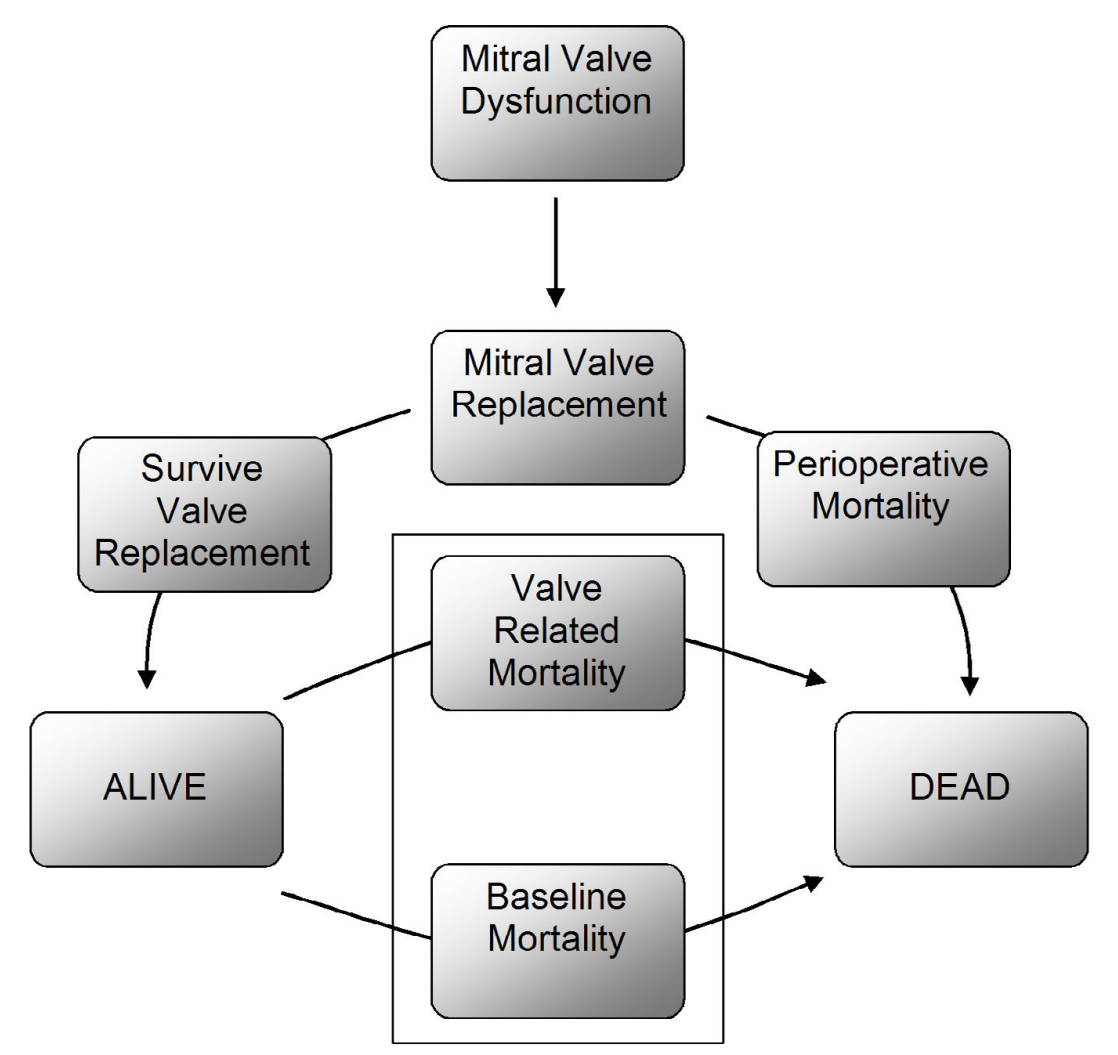
**Patients exist in either the "alive" or "dead" state.** A patient can remain in either the "alive" state, or can move from the "alive" to the "dead" state at the end of each cycle. The probability of state transition is determined by valve-related mortality and baseline age and gender specific mortality. Whilst a patient exists in the "alive" state they accumulate an incremental utility payoff determined by the valve replacement technique used.

An overview of our microsimulation model is shown in Figure 1. In our model we combine baseline mortality [31], with the mortality [32] and HRQoL associated with MVR in order to estimate long-term survival and Quality-Adjusted Life Years (QALY). QALY are a product of the survival and HRQoL (or Utility) that a patient experiences over a defined time horizon.

The probability of moving between states at the end of each cycle was randomly drawn from assigned probability distributions (Table 1). Each microsimulation was repeated 1000 times to generate a "virtual" cohort of 1000 patients.

**Table 1:** Summary of Model Parameters

MODEL PARAMETER	VALUE		RANGE/SD		DISTRIBUTION
Valve-Related Mortality Hazard Ratio 1 Year [5]	0.1084	0.0782	-	0.2049	Triangular
Valve-Related Mortality Hazard Ratio 5 Year [5]	0.0416	0.0197	-	0.0833	Triangular
Overall Survival without Preservation (62 year old cohort) [32]					
Year 1	87.4%				
Year 2	84.3%				
Year 3	81.3%				
Year 4	77.5%				
Year 5	75.0%				
Overall Survival without Preservation (82 year old cohort) [32]					
Year 1	79.8%				
Year 2	65.9%				
Year 3	64.1%				
Year 4	58.1%				
Year 5	40.7%				
Baseline Mortality [31]			GAD Life Tables		
Postoperative Utility Without Preservation					
Age 60–69	0.6668		0.1410		Normal
Age 70–79	0.6292		0.1330		Normal
Age 80+	0.6058		0.1281		Normal
Postoperative Utility With Preservation					
Age 60–69	0.7345		0.0660		Normal
Age 70–79	0.6931		0.0622		Normal
Age 80+	0.6673		0.0599		Normal
Discount Rate [44]	0.0350	0.0000	-	0.0600	Triangular

Analysis was performed over a 10 year time horizon, with one year cycles. Effects were discounted at 3.5% and a range of 0–6% was used for sensitivity analysis according to National Institute of Health and Clinical Excellence (NICE), guidelines on health technology assessment [44]. The analysis was performed using decision analytical software (TreeAge-Pro TM, TreeAge, Williamstown, Massachusetts, USA).

We validated the model structure by comparing overall survival in the non-preservation group at 5 years, with empirical data obtained from the UKHVR [32]. We chose to use the UKHVR rather than data from randomised controlled trials as we felt it was important to ensure that the results of our study should be applicable to every-day cardiothoracic practice in the United Kingdom.

### Investigating uncertainty: probabilistic sensitivity analysis and alternative analysis

There is an element of uncertainty associated with all attempts to consider long-term outcomes following surgical interventions. In Monte-Carlo simulation the modelling was repeated 1000 times for each cohort to generate probabilistic estimations of the combined effect of model parameter uncertainty [34].

Alternative analysis was performed to investigate the sensitivity of our results to patient age and the assumptions that we made about long-term valve-related mortality.

### Model parameters: survival data

The microsimulation model combines population baseline mortality [31] with valve-related mortality [32] in order to estimate overall survival for patients undergoing MVR.

The baseline population mortality was obtained from Government Actuarial Department (GAD) life tables [31] for a 62-year-old male cohort, as this is the mean age of patients undergoing mitral valve replacement in the United Kingdom [47].

Valve-related mortality for the non-preservation group was calculated by removing baseline population mortality from overall survival of the 14,148 patients recorded in the UKHVR who underwent MVR [32]. Valve-related mortality in the preservation group was calculated using estimates of valve-related mortality in the non-preservation group [32] and hazard ratios comparing preservation and non-preservation techniques. These hazard ratios were obtained from the meta-analysis of 2933 patients from 17 randomised and non-randomised studies by Athanasiou et al [5] which reported hazard ratios at 1 and 5 years. Hazard ratios and confidence intervals for years 2, 3 and 4 where calculated using linear interpolation. After 5 years the valve-related hazard was assumed to remain constant. We recognise the limitations of this assumption, however because of limited data we were unable to do otherwise. To investigate the sensitivity of our results to this assumption we performed an alternative analysis in which we assumed that there was no valve-related mortality after year 5.

As there is a trend to perform valve replacement in the United Kingdom in increasingly elderly patients [48] the effect of patient age on long-term outcomes following valve replacement was investigated in an 82 year old cohort using estimates of baseline mortality, obtained from GAD Life tables [31], and valve-related mortality obtained from in the same way as in the base case from the 86 patients aged over 80 who underwent MVR in the UKHVR [32].

### Model parameters: HRQoL

As there are no published studies comparing HRQoL (utility) following conventional MVR and SAP, HRQoL following MVR was estimated using data on post-operative New York Heart Association (NYHA) class. 8 studies, including non-randomised studies, reported postoperative NYHA class [17-24]. Data was not used from 3 [22-24] as the study groups where not matched preoperatively according to NYHA class and consequently we could not ensure postoperative differences in NYHA class were due to a treatment effect.

In order to calculate the utility of patients in each of the cohorts (Table 1) we used published data on the utility decrement suffered by patients in each NYHA class [49], EuroQol [50] age-specific data for baseline utility in the United Kingdom, together with the number of patients in each NYHA class extracted from the included studies [17-21]. Calculated age-specific utility values are shown in Table 2 together with data from the 677 patients used to calculate them.

**Table 2:** Utility Parameters used in the Model

	**Number of Patients [17–21]**		**% Utility**	**HRQoL**	**Age 30–39**		**Age 40–49**		**Age 50–59**		**Age 60–69**		**Age 70–79**		**Age 80+**	
**NYHA Class**	**With Preservation**	**Without Preservation**	**Decrement [49]**	**(Utility)**	**Mean**	**SD**	**Mean**	**SD**	**Mean**	**SD**	**Mean**	**SD**	**Mean**	**SD**	**Mean**	**SD**
	
0	0	0	0.00													
1	207	194	0.03	UK Baseline [50]	0.86		0.85		0.81		0.80		0.75		0.73	
2	74	126	0.20													
3	5	47	0.35	With Preservation	0.79	0.07	0.78	0.07	0.75	0.07	0.73	0.07	0.69	0.06	0.67	0.06
4	0	24	0.70													
				Without Preservation	0.72	0.15	0.71	0.15	0.68	0.14	0.67	0.14	0.63	0.13	0.61	0.13
														
**TOTAL**	286	391														

## Results

### Survival

**Figure 2:**
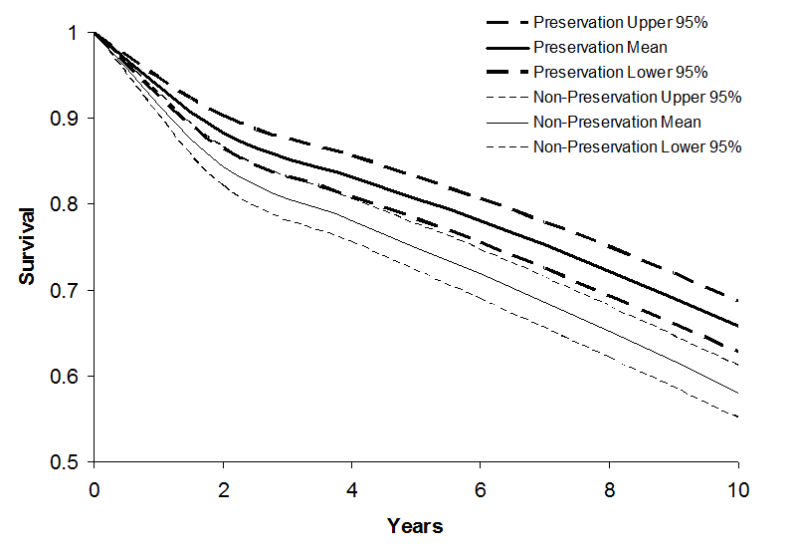
10-year survival curves illustrating survival following SAP and conventional MVR.

In the base case analysis 10-year survival was significantly improved following SAP (65.7% SD 1.5%, compared with 58.1% SD 1.6% at 10 years) with a difference in survival between conventional MVR and SAP of 7.6% (SD 0.9%) at 10 years (Figure 2). 5-year survival in the non-preservation group was 75.1% SD 1.4%, not significantly different to the empirical estimates given by the UKHVR [32] (73.8%), thus supporting the validity of our model structure.

### QALY

**Figure 3:**
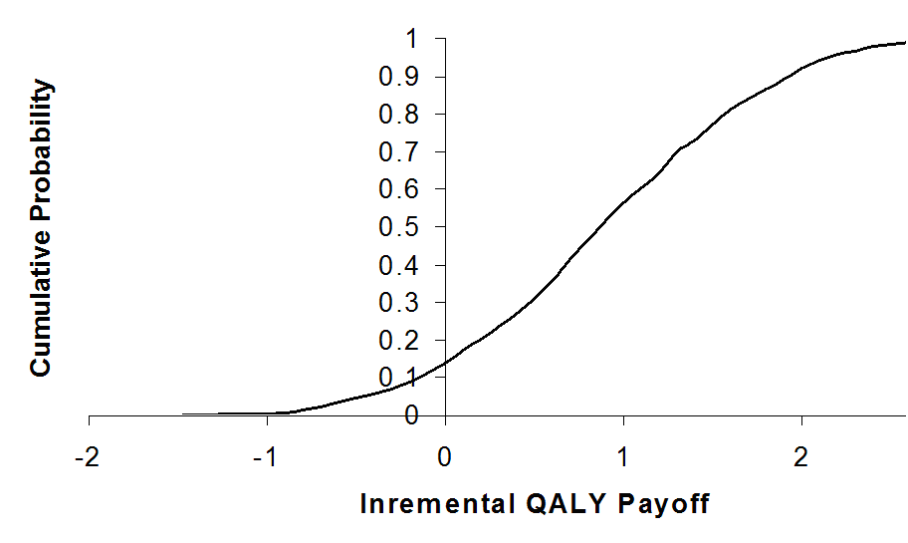
Cumulative probability distribution of incremental QALY payoffs for SAP compared to conventional MVR.

Over a 10-year period the mean QALY payoff was 5.61 QALY, (SD 0.07 QALY), without SAP, and 6.54 QALY (SD 0.07 QALY) with SAP. This represents an incremental QALY gain of 0.92 QALY (SD 0.04 QALY) for SAP compared with conventional MVR (Figure 3).

### Sensitivity analysis

Probabilistic sensitivity analyses suggests that survival at 10 years is superior following SAP compared to conventional MVR with 100% certainty, and that the QALY payoff is superior with 86.3% certainty.

### Alternative analysis

Absolute (15.4% SD 1.1% following SAP compared to 14.5% SD 1.1% following conventional repair) and incremental survival (0.09% SD 0.03%) were predictably higher in the base case compared to the elderly patient cohort, given the higher baseline mortality in the more elderly cohort. Probabilistic sensitivity analysis still suggested that survival was superior following SAP with 100% certainty. In the elderly the QALY payoff was 4.29 QALY (0.07 QALY) in the preservation group and 3.76 QALY (0.08 QALY) in the conventional group. Probabilistic sensitivity analysis suggested that the QALY payoff was superior following SAP with 73.1% certainty.

Survival (69.4% SD 1.5% following SAP, compared to 64.5% SD 1.5% following conventional repair) and QALY payoff (6.61 QALY, SD 0.07 QALY following SAP, compared to 5.72 QALY, SD 0.07 QALY following conventional repair) were higher in the alternative analysis compared to the base case. The incremental outcomes, however, were similar (4.9% SD 0.7%, 0.89 QALY SD 0.04 QALY), suggesting that our findings were not sensitive to the assumptions we made about valve-related mortality.

## Discussion

Our results suggest that patients survive longer following MVR with SAP (65.7% SD 1.5%, compared with 58.1% SD 1.6% at 10 years). Whilst this represents a significant improvement in survival (7.6% SD 0.9%) it is entirely consistent with published empirical data, arguably even underestimating the benefits of SAP [25]. Our study also suggests that these improvements in survival remain significant irrespective of patient age. Furthermore, this study estimates long-term improvements in HRQoL following SAP, suggesting that in our base case there was an incremental QALY gain of 0.92 QALY (SD 0.04 QALY) over 10 years. Probabilistic sensitivity analysis and alternative analysis suggest that theses results are not sensitive to uncertainty associated with the model parameters, structure or patient age.

The main cause of death after MVR is myocardial failure [51]. Several animal and human echocardiographic physiological studies have shown better maintenance of left ventricular function following SAP [6-8,51-55]. It is suggested that this is because papillary muscles are important to left ventricular contraction as they draw the mitral ring toward the apex, causing shortening of the long axis and spherity of the chamber, thereby contributing to better ejection of blood [56]. Despite existing evidence suggesting that SAP reduces morbidity and mortality [9-17] the sub-valvular apparatus is not always preserved often because it is not possible to preserve them. It is argued that the preserved sub-valvular apparatus prevent an adequately sized prosthetic valve from being used, and cause left ventricular outflow tract obstruction, by interfering with prosthetic valve function [26,57-60]. Whilst techniques to eliminate outflow tract obstruction following sub-valvular apparatus preservation have been described [27], it is reported that some of the preservation techniques cause alteration of the left ventricular geometry, causing rupture of the papillary muscles, systemic embolization and dehiscence of the mitral leaflets from their transposed positions as well as increasing ischemic time [26,56-60]. Finally SAP is often not technically possible because of active endocarditis, anatomical or pathophysiological considerations [5,28,61].

However, despite these concerns the evidence is clear. This study suggests that HRQoL is improved following SAP. It supports previously published data sets and meta-analytical data [5] which demonstrate that survival is significantly improved following SAP and represents further evidence that when technically feasible SAP should be routinely performed.

### Study limitations

The findings of our microsimulation are weakened by a lack of empirical data, particularly randomised data, in several important areas. Firstly, in this study HRQoL was calculated from the surrogate outcome, NYHA heart failure class because no empirical data was available on the association between SAP and HRQoL. Whilst NYHA has been shown to be associated with HRQoL [49], this methodology has not been validated in patients after MVR. Furthermore, we made the assumption that NYHA class remained constant during the follow-up period, and only accounted for differing mortality in different NYHA classes indirectly through the higher mortality in patients in the conventional MVR group who also tended to have worse NYHA function. Finally in many cases we relied on data from non-randomised sources to populate our model as high-quality, relevant randomised data was not available.

We did not consider the effect of re-intervention because of the dependence of valve-life on the type of prosthesis used, and limited information on the rates of redo-MVR following different techniques. This is an important limitation as outcomes are worse following re-intervention [28]. We feel that its impact on our results, however, was minimal as the mortality associated with early valve failure and subsequent re-intervention has been accounted for in the valve-related mortality, and the time horizon of our analysis was limited to 10 years.

There are more general weaknesses associated with decision analytical techniques such as microsimulation, for example, an increasing tendency to accumulate modelling error as the time horizon increases, and a tendency to overly formalise or simplify problems [33,34]. We minimised the impact of accumulated modelling error by limiting our analysis to a 10-year horizon, and validated our model structure using empirical data.

Finally, because of an absence of empirical data on long-term HRQoL and survival we were unable to consider the relative efficacy of different SAP techniques, in particular bi-leaflet preservation. This is important as there is relevant evidence from randomised controlled trials, that bi-leaflet preservation may result in superior outcomes compared to other partial preservation techniques [29,62].

## Conclusion

Despite the assumptions that we where forced to make in our analysis because of insufficient data, this study supports previous evidence of the superior efficacy of SAP compared to conventional MVR. We have quantified benefits in both survival and QALY following SAP, and shown these results to be insensitive to uncertainty about model parameters or structure. Whilst further research clearly needs to be conducted to establish whether complete SAP further improves results, this study suggest that SAP may improve survival and HRQoL following MVR.

## Abbreviations

GAD: Government Actuary's Department; HRQoL: Health Related Quality of Life; MVR: Mitral Valve Replacement; NICE: National Institute of Health and Clinical Excellence; NYHA: New York Heart Association; QALY: Quality Adjusted Life Years; SD: Standard Deviation: SVP: Sub-valvular Apparatus Preservation; UKHVR: United Kingdom Heart Valve Register.

## Authors' contributions

CR was responsible for study design, statistical analysis, data interpretation, and manuscript drafting. JH and AC were responsible for manuscript drafting, critical editing and revision for important intellectual content. PT and BM were responsible for data collection, data extraction analysis and interpretation. FS was responsible for data extraction analysis and critical statistical analysis. AD and FCW were responsible for providing important intellectual content throughout the manuscript's production and for final approval of the version to be published. TA is the guarantor for this paper and accepts full responsibility for the work and/or the conduct of the study. His involvement was critical to every phase of this work. He had access to the data, and controlled the decision to publish. All authors read and approved the final manuscript.
